# Targeting Amyloid-β Proteins as Potential Alzheimer’s Disease Therapeutics: Anti-Amyloid Drug Discovery, Emerging Therapeutics, Clinical Trials and Implications for Public Health

**DOI:** 10.3390/ph18111731

**Published:** 2025-11-14

**Authors:** Asaad Abdulrahman Abduljawad, Khadijah B. Alkinani, Aysha Zaakan, Abeer S. AlGhamdi, Alashary Adam Eisa Hamdoon, Batool H. Alshanbari, Ahmed Abdullah Alshehri, Badria Bakheet Alluhaybi, Shahad Othman Ibrahim Alqashi, Ryan Abdulrahman Abduljawad

**Affiliations:** 1 Department of Public Health, College of Health Sciences, Umm Al-Qura University, Makkah 21955, Saudi Arabia; kbknani@uqu.edu.sa (K.B.A.); aahamdoon@uqu.edu.sa (A.A.E.H.);; 2 Department of Chemistry, Faculty of Sciences, Taibah University, Madinah 42353, Saudi Arabia; azaakan@taibahu.edu.sa; 3 Department of Biochemistry, Faculty of Science, King Abdulaziz University, Jeddah 21589, Saudi Arabia; aalghamdi4218@stu.kau.edu.sa; 4 Health Management and Medical Informatics Department, College of Health Sciences, Umm Al-Qura University, Makkah 21955, Saudi Arabia; aashehri@uqu.edu.sa; 5 Medical Complex at the Public Services Center in Alshumaisy—Ministry of Health, Mecca 24231, Saudi Arabia; 6 Public Health Department, King Abdulaziz Airport, Ministry of Health, Jeddah 21442, Saudi Arabia; 7 King Salman Bin Abdulaziz Hospital, Ministry of Health, Riyadh 12769, Saudi Arabia

**Keywords:** Alzheimer’s disease, public health, anti-amyloid therapeutics, monoclonal antibodies, clinical trials, drug discovery, innovative approaches

## Abstract

Alzheimer’s disease (AD), a neurodegenerative disorder of the aging brain, is associated with behavioral and cognitive issues and poses a huge burden on the global health care system. One of the key features of AD is the deposition of abnormal proteins called amyloid-beta (Aβ) in the brain, causing inflammatory changes, oxidative stress, and neuronal loss. Recent advancements in the anti-Aβ therapies have considerably improved the management of AD, resulting in better clinical outcomes for patients and caregivers. This review offers an inclusive update on current drug discovery efforts, innovative approaches, and ongoing clinical trials targeting Aβ, a key player in AD pathogenesis. We have evaluated the most recent developments in monoclonal antibodies, including aducanumab (discontinued November 2024), lecanemab, and donanemab, emerging therapeutic options, as well as emerging strategies such as tau-targeting therapies, gene therapy, and small molecule inhibitors. Moreover, we highlighted the challenges and opportunities in AD research, including the need for early diagnosis, personalized medicine, and combination therapies. Our review will offer a concise and informative overview of the current landscape and future directions in anti-Aβ therapeutics for AD, shedding light on potential treatments and prospects for improving patient outcomes.

## 1. Introduction

Alzheimer’s disease (AD) is a neurodegenerative disease of the aging brain associated with cognitive decline, amnesia, and fluctuations in behavior and personality [1]. According to the Alzheimer’s Association 2020 reports, it is the widespread cause of dementia in the old age population and accounts for about 60–80 percent cases [2]. The AD pathology is multi-factorial, including an abnormal accumulation of amyloid peptides called Aβ inside the brain causing initiation of the inflammatory processes and damages to the neuronal cells [3]. The World Health Organization (WHO) reported that about 55 million individuals suffer from dementia worldwide, among which AD account for 60–70% of these cases [4]. Recent prevalence data suggests that the incidence of AD will be tripled by 2025 and will thus affect more than 152 million people [5,6]. The USA Centers for Disease Control and Prevention (CDC) reported that about 5.8 million individuals are suffering from AD, which is expected to increase to 13.8 million by 2050 [7,8]. The disease has put a substantial burden on the health care system, with a predicted cost of 2.8 trillion USD by 2030 [9]. Most of the clinically approved drugs have limited efficacy as they only relieve the symptomology. These include cholinesterase inhibitors like galantamine, donepezil, and rivastigmine. These agents work by inhibiting the ACh metabolizing enzymes (cholinesterase’s), reinstate the cholinergic activity, improve cognitive performance, and relieve the disease symptoms [10]. Another drug, memantine, is an NMDA receptor antagonist which blocks the abnormal activity of glutamate and thus improves cognitive performance [11]. Two clinically approved monoclonal antibodies, including lecanemab and aducanumab (now discontinued), are aimed to target the Aβ in the brain and reduce their load, thereby hampering the progression of AD [12,13]. Yet, there is an unrelenting need for the discovery of disease-modifying agents which can halt or reverse the progression of AD. Ongoing research emphasizes targeting amyloid-β and tau, as well as exploring other potential therapeutic approaches [14,15].

According to the amyloid hypothesis, the accumulation of Aβ is among the key drivers in AD pathology and is among the cornerstone of the anti-AD research and drug discovery [3]. Yet this hypothesis has several limitations including the complexity of the disease, inadequate success of the anti-Aβ therapies as well as the likelihood that the amyloid accumulation may possibly be a symptom, rather than the actual cause, of the disease [16,17]. Regardless of these challenges, the Aβ remains a key therapeutic target owing to its intricate interplay with tau proteins, which contributes to the AD pathology [18]. The interface between Aβ and tau is believed to assist the creation of neurofibrillary tangles (NFTs) and thus promote the progression of disease [19].

The role of Aβ in the development of AD is also supported by data from genetic studies. For instance, mutations in the genes relevant to the production of Aβ, PSEN1, PSEN2, and APP cause familial AD [20]. Mutations in these genes cause the excessive production and deposition of Aβ, which highlights its critical role in the pathology of AD. Moreover, studies suggest that timely anti-Aβ interventions may help avert or slow down the progression of AD. Anti-Aβ therapeutics are reported to reduce the burden of Aβ in the brain and hamper cognitive decline in patients with mild-to-moderate AD [21,22]. Despite several limitations and challenges, the Aβ remains an important target for the discovery of anti-AD drugs.

The accumulation of the Aβ triggers a cascade of downstream events which contribute to the progression of AD. Among the key mechanisms of Aβ-mediated neurotoxicity include oxidative stress caused by the excessive liberation of ROS by Aβ, causing an imbalance between the antioxidant and pro-oxidant mechanisms [23]. The free radicals cause damage to the neuronal components, including DNA, lipids, and proteins, which lead to their death and neuronal dysfunctions. The Aβ also interfere with the mitochondrial function which hampers the production of ATP, and causes excessive production of ROS and overall disruption of mitochondrial dynamics [24]. The mitochondrial dysfunction is among the key hallmarks of AD and thus Aβ-induced excessive production of free radicals contribute to mitochondrial damages and contribute to AD progression. Moreover, the accumulation of Aβ also triggers neuroinflammation which causes astrocytes and microglial activation, leading to the release of pro-inflammatory cytokines [25]. The release of these inflammatory cytokines worsens the Aβ toxicity and further contributes to the neuronal damages and progression of disease. The interplay among these mechanisms is highly multifaceted, and Aβ toxicity is known to interfere with calcium homeostasis, cause the activation of apoptotic pathways, and lead to synaptic dysfunctions [26].

## 2. Alzheimer’s Disease Pathogenesis

### 2.1. Amyloid-β (Aβ) Plaques

The pathogenesis aspects of AD include the presence of Aβ plaques, NFTs, oxidative stress, and neuroinflammation. The Aβ plaques comprise beta amyloid peptides deposits which accumulate in the brain and lead to neuronal damages and death [27]. These peptides are produced by the enzymatic cleavage of the amyloid precursor protein (APP) via the sequential catalytic effect of enzymes including beta-secretase and gamma-secretase enzymes [28]. The beta peptides are insoluble and gradually accumulate in the brain. These peptides are highly neurotoxic as they disrupt mitochondrial function, and set free excessive free radicals which attack neuronal cells, initiating their damage [29,30]. The Aβ peptides mediate their neurotoxic effects via the induction of oxidative stress and neuroinflammation and increases phosphorylation of tau proteins as well as through direct toxicity mechanisms via the activation of cell death pathways. Normally the brain clears the Aβ peptides using various mechanisms including enzymatic degradation using insulin-degrading enzyme (IDE), neprilysin, and uptake through astrocytes and microglia [31]. However, any dysregulation in these mechanisms lead to Aβ accretion in the brain leading to AD pathology [32]. Understanding the extremely complex Aβ pathology may help researchers to develop novel therapeutic strategies for the discovery of agents which can mitigate its toxic effects. Some of the current strategies include anti-Aβ immunotherapeutics and small molecules targeting Aβ peptides.

### 2.2. Neurofibrillary Tangles (NFTs)

Another hallmark of the AD pathogenesis is the presence of insoluble clumps of highly phosphorylated tau proteins called NFTs [33]. Normally the neuronal microtubules are stabilized and regulated functionally by tau proteins; however, in AD patients these proteins become highly phosphorylated, which causes their segregation from the microtubules and accumulation in the form of NFTS. Various enzymes including glycogen synthase kinase-3β (GSK-3β) and cyclin-dependent kinase 5 (CDK5) mediate these processes [33]. The NFTs contribute to neuronal damages via different mechanisms, including oxidative stress, neuroinflammation, and disruption of microtubules [34]. The link between the Aβ peptides and NFTs is highly complex, and the Aβ through the hyper-phosphorylation of tau proteins contribute to the production of NFTs [34]. Research directed towards understanding the molecular mechanisms of NFTs pathogenesis might help in the discovery of agents which increase the microtubules stability and target tau proteins.

### 2.3. Neuroinflammatory Processes in AD

Neuroinflammation is among the key drivers of AD pathology, primarily mediated by microglial and astrocytes activation causing the release of pro-inflammatory cytokines and chemokine’s [35]. These inflammatory mediators initiate neuronal damages and contribute to the disease progression. In AD patients, the resident immune cells in the brain called microglia are activated, which contribute to neuroinflammation by the release of pro-inflammatory mediators [35]. Likewise, the astrocytes contribute to the regulation of neurotransmitters and maintenance of the BBB normally [36]. In AD patients, once activated, cause the release chemokine’s and cytokines including TNF-α, MCP-1, and IL-1β, which exacerbates the neuronal damages [36]. Up-regulation of these inflammatory processes and the release of mediators are linked with Aβ and NFTs mediated neuroinflammatory damages.

### 2.4. Oxidative Stress

Free radicals induced oxidative stress is another key pathological aspect of AD, whereby an imbalance occurs between the production of free radicals and the neutralization by brain antioxidant mechanisms [37]. The free radicals readily attack various components of the neuronal cells including DNA, proteins, and lipids which cause their damage and ultimately result in cell death [38]. The Aβ-mediated mitochondrial dysfunction and microglial activation are mainly responsible for the excessive liberation of free radicals in AD patients. Thus oxidative stress promotes neuroinflammation, damages cellular components, and disrupts the vital cellular processes [39]. The interplay between oxidative stress and Aβ and NFTs is complex as both Aβ and NFTs contribute to oxidative stress and, conversely, the oxidative stress contributes to the formation Aβ and NFTs [40]. The use of antioxidants and other therapeutic strategies directed towards the discovery of mitochondrial targeted therapies might be of considerable clinical significance in the therapeutic management of AD.

### 2.5. Potential Therapeutic Targets in AD

Being a highly complex and multifactorial neurodegenerative disease, an understanding of the molecular mechanisms of AD is extremely important for the development of safe and effective therapeutic agents [41]. Various potential targets have been identified so far which include targeting Aβ production and clearance, tau pathology, oxidative stress management, and control over neuroinflammatory damages. Inhibitors of the key enzymes (beta-secretase, gamma-secretase) implicated in the production of Aβ have been extensively explored. Likewise, enzymes directed towards clearance of Aβ like neprilysin and anti-Aβ immunotherapeutics have also revealed considerable promise during the clinical trials [41]. Similarly, tau targeting therapies including tau immunotherapeutics and tau kinase inhibitors are undergoing thorough research for the discovery of anti-tau agents [42]. Coping with oxidative stress, neuroinflammation, and modulators of mitochondrial dysfunction are among the key targets for the discovery of more effective agents against AD [43,44].

## 3. The Amyloid-Premise

### 3.1. Historical Background, Biochemical and Pathological Evidence

The pathological link between the amyloid deposition and AD was coined back in the 1990s by John Hardy and David Allsop, who anticipated that the deposition of Aβ is among the key events in the pathogenesis of AD [45]. The discovery of Aβ inside the senile plaques further strengthened the discovery, and, subsequently, mutations were identified in the APP gene which leads to familial AD. This hypothesis was supported by the discovery of Aβ in senile plaques [46] and the identification of mutations in the amyloid precursor protein (APP) gene that cause familial AD further supported the theory [47]. Evidence indicates that the Aβ accumulation in the brain activates a cascade of events which eventually harm the neurons and lead to their death [3]. The Aβ hypothesis evolved further with the passage of time, and research revealed highly complex details about the production, deposition, clearance, and association with AD [1]. In spite of challenges and polemics, the amyloid hypothesis remains a central focus of AD research, driving the development of potential therapeutic approaches [14].

The theory of Aβ was supported by biochemical and pathological evidence which include the buildup of Aβ peptides in the AD brain, which combine to form insoluble fibrils that are toxic to neurons [3]. The Aβ peptides are generated via the enzymatic breakdown of APP by BACE1 (β-secretase) and γ-secretases. The hallmark of AD is the presence of senile plaques which are composed of Aβ deposits, and their occurrence relates strongly with the severity of disease [46]. Moreover, the genetic mutations in APP, presenilin-1 (PSEN1) and presenilin-2 (PSEN2) genes, upsurge the production of Aβ which is a major pathological marker of familial AD [47]. These discoveries offer robust substantiation for the amyloid hypothesis, which remains a central focus of AD research and therapeutic development.

### 3.2. Role of Amyloid-β (Aβ) Peptides in AD

The abnormal deposition of Aβ peptides is among the key pathological features of AD. The production of Aβ is initiated by catalytic break-down of the APP via β-secretase and **γ**-secretase sequentially which is subsequently deposited in the neuronal cells causing oxidative stress, inflammation, and neuronal damage [48]. When the Aβ peptides clump together to form insoluble fibrils, they disrupt the neuronal plasticity and synaptic physiology and gradually lead to cognitive dysfunctions. The deposition of senile plaques consisting of amyloid deposits is among the key features of AD and correlates with the disease severity. Thus, therapeutic strategies are directed towards targeting Aβ production, their aggregation, and faster clearance to halt or slow the disease progression [49].

## 4. Clinically Approved Alzheimer’s Disease Therapeutics

The clinically approved AD therapeutics includes cholinesterase inhibitors like galantamine, donepezil, and rivastigmine [50,51]. These agents work by inhibiting the cholinesterase-mediated enzymatic degradation of the important neurotransmitter acetylcholine (Ach) [52]. By inhibition of these metabolizing enzymes, the ACH may remain for a prolonged time at the synaptic cholinergic receptors and mediate prolonged cholinergic activity, which provide relief from the symptoms and improve cognitive performance [53,54,55]. Among the approved cholinesterase inhibitors, the galantamine not only provide a selective and reversible inhibition of cholinesterase but also act as an allosteric potentiator of the nicotinic receptors (α4β2 and α7) of Ach [50]. This dual action of galantamine considerably contributes towards its therapeutic action in the management of AD [56]. The donepezil is an inhibitor of ACHE and rivastigmine is a pseudo-reversible inhibitor of both AChE/BChE enzymes [50]. Another drug, memantine, blocks the NMDA receptors’ mediated calcium channels and reduces the glutamate mediated neurotoxicity as well as neuronal dysfunctions [57]. Though glutamate plays a vital role in learning and memory, its excessive liberation may cause over-activation of NMDA receptors and may cause neuronal damage and death. Memantine helps protect the brain by regulating glutamate activity, which improves symptoms of AD. These agents are also used in combination for more useful outcomes [57].

The discovery of anti-Aβ therapeutics is considered a paradigm shift in the treatment of AD. In contrast to the traditional therapies which merely alleviate the cognitive decline and relieve the symptoms only, these therapies mainly target the disease’s underlying pathology [1]. The clinically approved monoclonal antibodies like lecanemab and aducanumab (discontinued) help to neutralize and reduce the Aβ load in the brain [12]. These disease modifying agents help to slow the disease progression and, by targeting the key driver of the disease pathology, may possibly transform the AD landscape and may improve the patient-related outcomes [58]. In anti-amyloid therapies, various molecules are in different stages of clinical trials, but no drug is approved except the Aβ targeting antibodies like aducanumab (approved for clinical use in June 2021 and discontinued November 2024) and lecanemab [12,13]. The manufacturer Biogen discontinued the production of aducanumab (Aduhelm) in November 2024. The manufacturer stated that the discontinuation of the product is not due to safety or efficacy issues but to reprioritize its resources towards lecanemab and the development of other new products [59]. Other therapeutic approaches include anti-tau agents, like AADVac1 and semorinemab as well as modulators of neuroinflammation including sargramostim and AL002 [60,61].

## 5. Amyloid-Targeting Therapies

### 5.1. Anti-Aβ Antibodies

**Figure 1:**
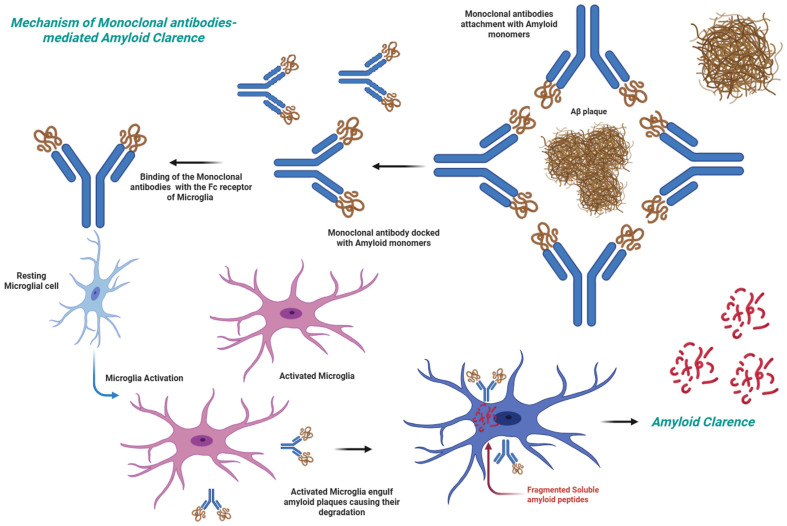
Monoclonal antibodies mediated clearance of Aβ therapeutic agent.

Among the most promising anti-amyloid therapies is the use of anti-Aβ antibodies, which help in reducing the amyloid load in the brain and hamper progression of the disease [13] (Figure 1). A more comprehensive overview of different types of monoclonal antibodies is provided in Table 1 and Section 5.

### 5.2. Clinically Approved Anti-Aβ Antibodies

Novel antibodies, some of which are approved for clinical use and others of which are in various stages of clinical phases, are of considerable significance in targeting the Aβ of AD patients.

#### 5.2.1. Lecanemab

Lecanemab, a monoclonal antibody, works by binding with soluble Aβ fibrils and large soluble Aβ aggregates which lead to neurotoxicity [12]. The suggested dose is 10 mg/kg body weight and administered via I/V route after every two weeks [62]. In clinical trials, lecanemab has revealed considerable efficacy in reducing the Aβ load and exhibited cognitive benefits in early stage AD patients [12]. The most common disadvantages associated with lecanemab include ARIAs and infusion-associated moderate reactions [63].

#### 5.2.2. Aducanumab

Aducanumab (discontinued November 2024) is able to selectively bind with the aggregated forms of Aβ, which help in reducing the burden of plaques and help to reduce cognitive decline [64]. During the phase-3 clinical trials, including EMERGE and ENGAGE trials, aducanumab exhibited satisfactory outcomes by achieving the primary endpoint whereas the EMERGE studies did not meet the primary endpoint outcomes [64]. During the long term and high dose studies, considerable benefits were observed in AD patients. Among the common side effects of the treatment include ARIA. The antibody was subsequently approved for clinical applications by the FDA in 2021 [65]. However, a subsequent analysis suggested potential benefits in patients with early Alzheimer’s disease.

#### 5.2.3. Donanemab

The monoclonal antibody donanemab, clinically approved by FDA with Kisunla^®^ brand, primarily targets Aβ plaques and thus facilitates their removal from the AD brain. This helps to hamper the disease progression and improve cognitive performance and behavioral outcomes [66]. The donanemab exhibited considerable efficacy during the clinical trials [67]. The antibodies mediate its therapeutic effect by binding with N-terminal truncated, pyroglutamate-modified Aβ 3(pE)-42, which is a particular type of the Aβ present in the plaques [68]. This antibody is also devoid of any major side effects except the ARIA [69].

## 6. Anti-Amyloid Antibodies in the Pipeline

### 6.1. Bapineuzumab

The humanized monoclonal antibody bapineuzumab is currently under phase-3 clinical trials which selectively target the Aβ and helps in its clearance from the brain [70]. Initial easements were satisfactory, but at phase-3 trials the target primary endpoints were not achieved, which indicate its deficiency to combat AD associated cognitive decline [71]. Yet, its benefits were more prominent in AD patients who are not carriers of APOEε4 allele [72].

### 6.2. Solanezumab

The monoclonal antibodies preparation solanezumab targets the Aβ plaques and works by reducing its burden in the brain [73]. During phase-3 trials, this antibody was safe and well tolerated. Unfortunately, it did not meet its primary end points during the trials, but secondary studies revealed its efficacy in reducing the progression of disease by 15–30% [74].

### 6.3. Gantenerumab

Another monoclonal antibody gantenerumab reduces the Aβ production and accumulation in the brain by enhancing its clearance [75]. Its efficacy and safety were evaluated in clinical trials like Scarlet RoAD and Marguerite RoAD in prodromal and mild AD patients [22]. Its safety and efficacy studies outcome are yet to be made public. The antibodies have shown a decline in AD biomarkers and associated cognitive decline, but is associated with common side effects including ARIA [76].

### 6.4. Crenezumab

The humanized monoclonal antibody crenezumab is an anti-Aβ preparation which help to reduce the Aβ proteins by inhibition of their formation and accumulation in the brain [77]. During the phase-3 trials (CREAD and CREAD-2), the antibodies did not achieve its primary endpoints, though its safety profile is up to the mark [78]. Further studies are underway to uncover its efficacy and associated effects in AD patients.

### 6.5. Remternetug

An anti-Aβ investigational monoclonal antibody remternetug was developed by Eli Lilly which, in the clinical trial NCT05463731 aimed to evaluate its safety and efficacy, indicated a faster and robust decline in Aβ content [79]. During the phase-1 trials, about 75% of the patients achieved amyloid clearance and the antibodies were devoid of any major safety concerns except the common side effects like ARIA. Further phase-3 trials are underway to evaluate its safety and efficacy in larger groups of patients [80].

### 6.6. ABBV-916

In phase-2 clinical trials (NCT05291234), the AbbVie developed anti-Aβ antibody ABBV-916 was evaluated for safety, efficacy, ADME, and pharmacodynamics properties. Results of the outcomes are yet to be made public and the trial is active until 2025, though not recruiting participants currently [81].

### 6.7. Trontinemab

The monoclonal antibody preparation trontinemab is a highly promising effort for the AD treatment, which utilizes Roche’s proprietary brain-shuttle technology for delivering the bi-specific antibodies across the BBB [82,83]. During the clinical trials, the antibody is reported to inhibit and reduce the Aβ load in the brain. During the phase 1n/11a trials, 28 weeks of treatment achieved about 91% amyloid PET negativity rate amongst the participants, and about 72% achieved deep amyloid clearance. The antibody preparation has a highly favorable safety profile with only minor imaging related abnormalities observed in <5% participants [82,83].

## 7. β-Secretase (BACE-1) and γ-Secretase Inhibitors

**Figure 2:**
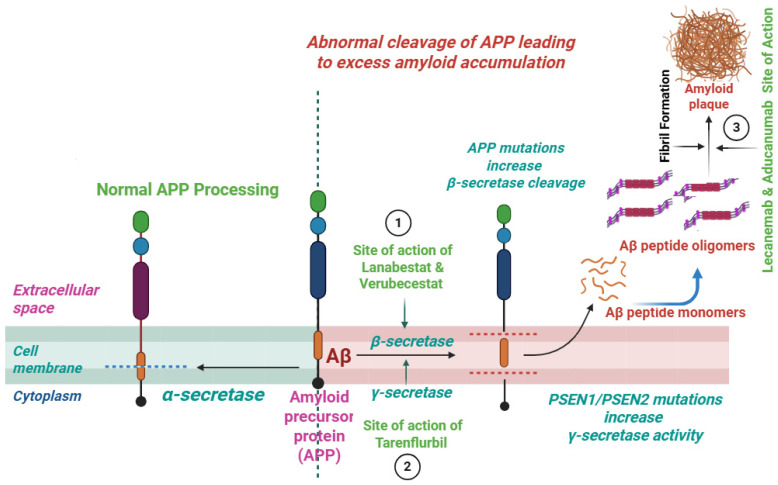
Site of action of BACE1 inhibitors, γ-secretases modulators, and Aβ aggregation inhibitors.

Inhibitors of the β-secretase enzyme decline the Aβ production via reduction in the enzymatic breakdown of APP and thus slow down the progression of AD [84] (Figure 2). Among the tested agents, lanabecestat and verubecestat have revealed promising results in the early phases of clinical trials. Yet in the advanced phases of clinical trials (phase-3) they were associated with efficacy issues as well as associated side effects [85,86]. More research is required to optimize BACE1 inhibitors and increase their efficacy and safety profiles.

The modulators of the γ-secretase enzyme reduce the load of Aβ by preserving the Notch signaling and thus reduce its production associated with neuro-cognitive issues. The bioactive agents inhibiting γ-secretase, including CHF5074 and tarenflurbil, selectively modulate the enzyme activity and reduce the production of Aβ_42_ without affecting Notch processing [87,88] (Figure 2). Though these agents have limited efficacy, recently developed agents have much improved potency and selectivity. The use of these modulators may offer a safer alternative to γ-secretase inhibitors, which can cause mechanism-based toxicities due to Notch inhibition.

Failure of both BACE1 and γ-secretase inhibitors during the phase-3 clinical trials indicate the highly complex pathological nature of AD and challenges associated with the production of Aβ. The off-target toxicity is among the major associated issues, as both BACE1 and γ-secretase have several substrates and play critical roles in various cellular processes [89,90]. The absence of the specificity of these agents may cause mechanism-based unwanted effects which can outweigh their beneficial effects. The failure of these agents suggests more detailed understanding of the disease pathology and discovery of more feasible targets and drug molecules. In this regard, combining multiple agents in a single formulation may target multiple disease targets and may be more effective in comparison to single target agents.

## 8. Inhibitors of the Aβ Aggregation

Another viable approach is the design of therapeutic agents aimed at inhibiting the aggregation of amyloid plaques. They act at various stages during the Aβ aggregation, like the initial step of oligomerization, subsequent fibril formation, and then stabilization of aggregate forms [91]. Natural flavonoids including EGCG and compounds like scyllo-inositol were found to inhibit these steps and have proven anti-aggregation potentials [92,93]. Moreover, peptide-based inhibitors were found to selectively bind with Aβ and inhibit its aggregation [94]. Therefore, via decline in the Aβ toxicity and accumulation, these agents might hamper the progression of disease and offer a potential therapeutic approach for AD treatment (Figure 2).

## 9. Other Innovative Approaches in Alzheimer’s Disease

### 9.1. Tau-Targeting Therapies

The tau-targeting strategies include the use of therapeutics capable of reducing the aggregation of tau proteins, tau hyper-phosphorylation, and the formation of NFTs which are the key pathological aspects of AD [95,96]. For instance, tideglusib and lithium inhibit GSK-3β, which subsequently inhibits tau hyper-phosphorylation [97,98]. Moreover, another therapeutic candidate, the LY3372689, which is in phase 2 clinical trials, inhibits O-GlcNAcase (OGA) and reduces tau phosphorylation. The immunotherapies including active vaccination via AADvac1 and passive vaccination via bepranemab, JNJ-63733657, and E2814 antibodies have also revealed considerable efficacy in reducing tau [96]. Moreover, the use of antisense oligonucleotides (ASOs) including BIIB080 has revealed a promise in reducing tau expression via inhibition of the tau mRNA translation. Despite various challenges, the tau-targeting therapies hold promise for treating AD, with several approaches being recognized for the modification of tau pathology.

### 9.2. Immunotherapies

The immunotherapeutic strategies in AD include the modification of the course of the disease by targeting Aβ and NFTs via the use of active and passive immunization tools [99]. Administration of vaccines can provoke the immune system of the patients to generate anti-Aβ and anti-tau antibodies [100]. In passive immunization, purified monoclonal antibodies are administered which target these abnormal proteins and help in their removal from the brain [101]. The immunotherapies including aducanumab (discontinued November 2024), lecanemab, and bapineuzumab specifically target Aβ, and vaccines including AN1792 and Protollin help in the removal of these abnormal proteins. However, the use of these agents is associated with various issues including meningo-encephalitis, vasogenic edema, and limited bioavailability across the BBB, which limit its therapeutic efficacy. Various prospective immunotherapeutics are in different stages of clinical trials and have considerable efficacy in reducing the Aβ aggregation and improvement in the management of clinical symptomology [100,101]. Yet, further research is required for the optimization of design and delivery for more effective therapeutic outcomes.

### 9.3. Stem Cell Therapies

The use of stem cell therapy is another promising approach in the treatment of AD and offers various advantages as compared to traditional drug therapies [102]. Various stem cells like neural stem cells (NSCs), embryonic stem cells (ESCs), mesenchymal stem cells (MSCs), and induced pluripotent stem cells (iPSCs) which were found useful in reducing neuroinflammation, have improved neuronal regeneration as well as cognitive performance in models of AD [103,104,105]. Of particular interest are the MSCs which have revealed considerable accessibility and differentiating potential, with MSC-derived exosomes signifying potential in controlling neuroinflammation and better cognitive performance [106,107]. The stem cells based therapeutic approach has limited data availability from the clinical trials and is associated with side effects which necessitate further studies for better therapeutic outcomes.

### 9.4. Gene Therapies

Gene therapies are an important tool which enables researchers to replace or modify unwanted genes implicated in AD and replace them with alternative genes of interest. Among the gene editing tools is the use of the CRISPR/Cas9 technique which can edit genes, like APP, PSEN1, and PSEN2, involved in the production of Aβ [91]. Another tool is the use of therapeutic gene delivery like neprilysin or IDE which help in the degradation of Aβ [108]. Another tool is the use of therapeutic genes delivery like neprilysin or IDE which help in the degradation of Aβ [109].

### 9.5. Neutrophins-Based Gene Therapies

Other approaches include the neutrophins-based gene therapy for the neurotrophic factors like BDNF and NGF which play an important role in neuronal growth and survival [110]. Using this approach, genes that encode neurotrophic factors (BDNF, NGF) are delivered to the brain using viral vectors [111]. Studies revealed that by increasing the brain neurotrophins levels, these agents promote the survival of neurons and enhance the cognitive performance of AD patients [112]. Neurotrophins help in maintaining the synaptic plasticity and its depletion is reported to cause neuroinflammation, accumulation of Aβ, and tau proteins hyperphosphorylation [113]. The delivery of neurotrophic encoding genes to the entorhinal cortex reduces the accumulation of Aβ and improves cognitive performance. The NGF delivery to the brain stimulates the survival of cholinergic neurons and improves cognitive performance in pre-clinical trials and clinical studies [114]. Another promising candidate for the neurotrophic gene therapy is the delivery of NF-α1, which enhances synaptic plasticity, reduces neuroinflammation and promotes survival of neurons [115]. While challenges remain, including crossing the blood–brain barrier and ensuring safety and efficacy, gene therapy holds potential as a disease-modifying treatment for AD.

### 9.6. Epigenetic Therapies

The epigenetic approaches in AD use gene expression modification thus mitigate the pathology of disease. The histone deacetylase inhibitors (HDACis) like sodium butyrate and valproic acid exhibited potentials in decreasing Aβ and tau pathology which improve synaptic plasticity and cognitive performance [101,116]. Other options in epigenetic research include the use of DNA methyltransferase inhibitors and histone acetyltransferase activators which can target multiple pathways and offer a more inclusive approach for the modification of disease.

### 9.7. Multi-Target Approaches

This approach uses multiple therapies by targeting multiple pathways and mechanisms underlying the disease simultaneously to modulate disease progression [117]. After analysis of the disease complexity, this approach involves targeting several factors like Aβ, neuroinflammation, tau, and oxidative stress using a single drug candidate. For targeting multiple aspects of the disease, molecules effective on various targets or combinations of molecules in a single dosage form are utilized [118,119]. For instance, in combination therapies, cholinesterase inhibitors, memantine, anti-Aβ agents, and natural bioactive molecules like curcumin and resveratrol which exhibit multi-target efficacy are combined [120].

### 9.8. Digital Therapeutics

For the therapeutic management of AD, the digital therapeutics (DTx) offer a promising approach by providing evidence-based interventions driven using various software. These digital health applications can exploit various tools including computers, mobiles, videogames, and sensors which can help in the prevention, management, and treatment of AD. Various approaches like cognitive stimulation, music-based therapies, and virtual reality are some of the DTx which are evaluated for the management of AD. The music-based DTx, particularly, has revealed potential in controlling anxiety and agitation and anxiety among AD patients by down-regulating the autonomic arousal [121]. Though the regulatory frameworks for using DTx are in infancy, the FDA’s Break-through Device Program might be useful to speed up the process of DTx development for the management of AD. Yet further research is required for the complete recognition of the risks and benefits associated with the use of DTx for management of AD.

### 9.9. Nano-Enabled Anti-AD Therapeutics

Among the key challenges in the drug discovery against AD is the target bioavailability of the potential agents whereby the blood–brain barrier (BBB) poses a significant resistance against the entry of foreign molecules. To cope with these challenges, nano-based therapies have emerged as a potential approach which by using nano-carrier systems helps to traverse the delivery of therapeutic agents across the BBB [122,123]. Different nanoparticles (NPs) based dosage forms including liposomes, inorganic NPs, and polymeric NPs are reported to exhibit anti-Aβ potentials [124]. These NPs can be modified and engineered for achieving target specific drug delivery and reducing systemic side effects for better therapeutic outcomes. Studies have shown that nanoparticles can improve the delivery of anti-Aβ agents across the BBB, reducing the Aβ plaque burden and improving cognitive function in animal models of AD [3]. For example, liposomes conjugated with antibodies against Aβ have been shown to selectively target Aβ plaques in the brain and promote their clearance [4]. Similarly, polymeric nanoparticles loaded with anti-Aβ agents have been demonstrated to reduce Aβ plaque burden and improve cognitive function in animal models of AD [5]. Research outcomes indicated that nano-based carrier systems can effectively deliver anti-Aβ agents with better bioavailability profiles and less side effects [125].

## 10. Challenges and Implications for Public Health

Keeping in view the extremely complex pathological aspects of AD, including the pathways and mechanisms, the discovery and development of effective therapies against AD is a real challenge. One of the challenges is the bioavailability of the therapeutic agents against at the target sites, as the human BBB possesses considerable hurdles by limiting the delivery of therapeutic agents to the brain. Through the integration of biotechnological aspects to the anti-AD drug design, more effective and targeted drug discovery can be ensured. Development of biogenic nano-formulations of tested natural compounds and natural products might open new avenues for the drug discovery with better safety, efficacy, and bioavailability profiles. The heterogeneity of the disease makes it difficult for the researchers to develop a single therapeutic agent for all AD patients. Unavailability of highly reliable markers which predict the progression of the disease and patients’ response to the therapeutic agents is another challenge. Moreover, the therapeutic candidates are associated with considerable side effects as amyloid-related imaging abnormalities (ARIAs) were observed with the use of anti-Aβ agents. These also pose a question on the efficacy and safety of these therapeutic options. Appropriate recognition of these challenges is very crucial for the discovery and development of more effective and safe anti-AD agents.

The future perspective should be directed towards the exploration of agents which are effective on more than one pathological target of the disease or potential multi-target agents. The options may include the discovery of agents which exhibit anti-Aβ properties coupled with anti-tau potentials to act synergistically against a vital pathological aspect of the disease. The anti-inflammatory agents may couple with antioxidants to reduce oxidative stress induced neuronal inflammation and damage. The integration of lifestyle modification and effective cognitive training with the current therapeutic agents might offer better efficacy in the prevention and treatment of AD. Thus, targeting multiple mechanisms of AD may offer improved efficacy and better disease management, potentially leading to more effective options for AD treatment.

The concept of personalized medicine can be applied to the AD patients by tailoring the individual treatments based on the patient’s clinical, molecular, and genetic profiles. This might be possible via stratification of biological markers, identification of disease progression and sub-types, as well as genetic profiling like APOE status. Using the precision medicine strategies, the clinicians and researchers can help to develop targeted therapies which can specially address the mechanisms of disease in individual patients and might provide more effective and tailored therapeutic options against AD.

Using multi-faceted approaches like biological biomarkers based sub-grouping might be useful in the identification of sub-groups, disease heterogeneity, and the development of targeted therapies tailored for individual patient profiles. Evaluation of the genetic and molecular, as well as clinical, characteristics of individual patients, means the personalized therapeutic approach can be adopted for better therapeutic outcomes. Yet, the development or identification of more effective biological markers for better prognoses is a challenge due to the complexity of the disease. Recognizing and addressing this heterogeneity can offer more effective and personalized therapeutic remedies against AD.

## 11. Conclusions

AD is an extremely complex, multifactorial neurological disease having high global prevalence, and is associated with high costs and poses considerable challenges to the health care system as well as towards drug discovery and development. Among the disease’s key pathological aspects is the deposition of Aβ in the brain, which causes the release of excessive free radicals, affecting the mitochondrial function, inducing inflammation, and initiating degeneration of the nerve cells leading to impaired signal transmission and subsequent behavioral and neurochemical issues. Subsequently, efforts have been directed towards the identification of the molecular aspects of Aβ deposition, hampering its excessive production and facilitating its early removal from the brain. In this regard, efforts have been made to find small molecules which can inhibit enzymes involved in the production of Aβ and the development of antibodies directed to its clearance from the brain. Several anti-Aβ preparations have shown promise in the removal of Aβ, improvement in cognitive performance, and modification of the disease progression. We have tried to encapsulate the efforts made especially against the amyloid pathology of AD, the clinically approved drugs, innovative approaches, the drug discovery pipeline and the status of the anti-amyloid clinical trials. Some anti-Aβ antibodies are already approved by FDA for mild-to-moderate AD including lecanemab, aducanumab (discontinued November 2024), and donanemab, whereas numerous antibodies are in various phases of clinical trials for safety and efficacy.

Beside anti-Aβ therapeutics, focus is also directed on other innovative approaches including tau-targeting therapies, immunotherapies-based vaccines, stem cell therapies, gene therapies, epigenetic therapies, and digital therapies. Multi-target approaches are underway for the prevention and discovery of more effective therapies against AD. However, the future of AD management is reliant on using AI-based drug discovery approaches to find novel and more effective drug targets, combining agents effective on multi targets like anti-tau agents, anti-inflammatory agents, anti-Aβ agents, inhibitors of the enzyme involved in the disease pathology like cholinesterase’s, BACE1 inhibitors, and neurotrophic agents might give more convincing results. Combining drug therapies with lifestyle modifications and digital therapies might help to hamper the progression of disease and improve the patient’s quality of life. As research continues to unknot the complexities of AD, the discovery of more useful therapies will rely on collaborative efforts among researchers, clinicians, and industry stakeholders.

Eventually, the quest of innovative, more useful anti-Aβ therapeutics holds promise for humanizing the lives of individuals affected by AD and their families. Further research and investment are essential to achieving this goal and addressing the growing burden of this devastating disease. While challenges persist, like the intricacy of AD pathology and the necessity for early intervention, the progress made in anti-Aβ drug discovery is highly promising. Ongoing research and clinical trials are vital in determining the efficacy and safety of these treatments.

## Figures and Tables

**Table 1:** Summary of the anti-amyloid drug discovery pipeline.

	Purpose of the Therapy	Target	Mechanism of Action	Clinical Trials Stage/Status	Clinical Trial No.	Starting Date	Proposed Completion	**Sponsor**
Aducanumab	Disease modifying biological agent	Aβ	Monoclonal antibodies that target oligomers and Aβ plaques	Discontinued Nov. 2024	NCT04241068	Mar-2020	July-2024	Biogen
					NCT05310071	Jun-2022	Aug-2024	Biogen
Donanemab	Disease modifying biological agent	Aβ	Monoclonal antibodies explicitly against pyro-glutamate Aβ	Phase-3	NCT04437511	Jun-2020	April-2023	Eli Lilly
					NCT05026866	Aug-2021	Oct-2027	
					NCT05508789	Oct-2022	April-2027	
					NCT05738486	Feb-2023	Mar-2024	
Gantenerumab	Disease modifying biological agent	Aβ	Monoclonal antibodies against Aβ	Phase-3	NCT01760005	Dec-2012	Oct-2027	Washington Univ. School of Medicine
Lecanemab	Disease modifying biological agent	Aβ	Monoclonal antibodies against amyloid proto-fibrils & Aβ	Phase-3	NCT01760005	Dec-2012	Oct-2027	Washington University School of Medicine & Eisai Inc.
					NCT03887455	Mar-2019	Sep-2027	
					NCT04468659	Jul-2020	Oct-2027	
					NCT05269394	Dec-2021	Jul-2027	
Lecanemab	Disease modifying biological agent	Aβ	Monoclonal antibodies against amyloid proto-fibrils and Aβ	Phase-2	NCT01767311	Dec-2012	Feb-2025	Eisai Inc.
Remternetug	Disease modifying biological agent	Aβ	Monoclonal anti-Aβ antibodies	Phase-3	NCT05463731	Aug-2022	Oct-2025	Eli Lilly
Solanezumab	Disease modifying biological agent	Aβ	Monoclonal anti-Aβ antibodies	Phase-3	NCT01760005	Dec-2012	Oct-2027	Washington Univ. School of Medicine
ABBV-916	Disease modifying biological agent	Aβ	Anti-Aβ antibodies	Phase-2	NCT05291234	April-2023	Jun-2024	AbbVie
ACI-24.060	Disease modifying biological agent	Aβ	Active vaccination stimulating anti-Aβ antibodies	Phase-2	NCT05462106	Jun-2022	Jun-2026	AC Immune SA
ALZN002	Disease modifying biological agent	Aβ	Autologous Aβ mutant peptide pulsed dendritic cells	Phase-2	NCT05834296	Jul-2023	Mar-2028	Alzamend Neuro Inc.
APH-1105	Disease modifying small molecule	Aβ	α-secretase modulator	Phase-2	NCT03806478	Jun-2023	Sep-2024	Aphios
MIB-626	Disease modifying small molecule	Aβ	Sirtuin nicotinamide adenine dinucleotide stimulator to Increase α-secretase activity	Phase-2	NCT05040321	Dec-2021	April-2024	Brigham & women’s hospital
PRI-002	Disease modifying small molecule	Aβ	Interfere with Aβ42 oligomerization	Phase-2	NCT06182085	Dec-2023	April-2026	PRInnovation GmbH
Trontinemab	Disease modifying biological agent	Aβ	Anti-Aβ and anti-oligomers monoclonal antibodies	Phase-2	NCT04639050	Mar-2021	Sep-2027	Hoffmann-La Roche
Valiltramiprosate	Disease modifying small molecule	Aβ	Inhibitor of Aβ aggregation	Phase-2	NCT04693520	Sep-2020	Jul-2023	Alzheon Inc.
Varoglutamstat	Disease modifying small molecule	Aβ	Reduce the pyroglutamate Aβ formation via inhibition of glutaminyl-cyclase enzyme	Phase-2	NCT03919162	Nov-2021	Nov-2023	Vivoryon Therapeutics
					NCT04498650	Jul-2020	Jan-2024	
ALN-APP	Disease modifying biological agent	Aβ	RNAi to reduce APP and down-stream Aβ related events	Phase-1	NCT05231785	Feb-2022	Jul-2025	Alnylam Pharma
ALZ-101	Disease modifying biological agent	Aβ	Anti-Aβ Vaccine	Phase-1	NCT05328115	Sep-2021	Dec-2023	Alzinova-AB
AV-1959D	Disease modifying biological agent	Aβ	Anti-Aβ Vaccine	Phase-1	NCT05642429	Feb-2023	Feb-2026	Institute of Medicine
BMS-984923	Disease modifying small molecule	Aβ	Silent allosteric modulator of mGluR5	Phase-1	NCT05804383	Mar-2023	Oct-2024	Allyx Therapeutics
					NCT05817643	Jan-2023	Feb-2023	
Remternetug	Disease modifying biological agent	Aβ	Anti-Aβ monoclonal antibodies	Phase-1	NCT04451408	Jul-2020	Aug-2024	Eli Lilli
SHR-1707	Disease modifying biological agent	Aβ	Anti-Aβ monoclonal antibodies	Phase-1	NCT06114745	Jan-2024	Nov-2025	Atridia Pty Ltd.

## Data Availability

The original contributions presented in this study are included in the article. Further inquiries can be directed to the corresponding author.
